# Five-Year study on renal outcomes in biopsy-proven focal segmental glomerulosclerosis patients in Shiraz, Iran

**DOI:** 10.22088/cjim.15.3.519

**Published:** 2024-08-01

**Authors:** Maryam Pakfetrat, Leila Malekmakan, Mohamad Hossein Rezazadeh, Taraneh Tadayon, Maryam Bahmani, Amirali Nikoo

**Affiliations:** 1 Shiraz Nephro-Urology Research Center, Shiraz University of Medical Sciences, Shiraz, Iran; 2 Student at University of Toronto Scarborough, Ontario, Canada

**Keywords:** Focal segmental glomerulosclerosis, nephrotic syndrome, glomerular filtration rate, chronic kidney disease

## Abstract

**Background:**

Focal segmental glomerulosclerosis (FSGS) is a prevalent glomerular disease that often leads to nephrotic syndrome. It is characterized by consolidating a portion of the glomerular capillary tuft connected to Bowman's capsule. This retrospective cohort study aimed to determine the demographic characteristics, risk factors, and prognostic indicators associated with FSGS in Shiraz, Iran.

**Methods:**

The study included 53 primary FSGS patients aged over 18 years who were referred to clinics affiliated with Shiraz University of Medical Sciences. Data were collected through a comprehensive data-gathering sheet encompassing demographic information, medical history, laboratory test results, and histopathological findings. Statistical analysis was performed using SPSS 18, considering a significance level of p<0.05.

**Results:**

A five-year follow-up was conducted on the 53 patients, with the mean age of 41.0±13.3 years. The most common FSGS variants observed were "not otherwise specified" (NOS, 13.2%) and tip variant (7.5%). Older patients exhibited higher disease activity, whereas remission rates were higher among younger individuals (P=0.012). Patients achieving remission had lower creatinine and Pro/Cr ratios and higher glomerular filtration rates (p<0.05). Treatment involving a combination of corticosteroids and mycophenolate mofetil showed a significant correlation with remission (P=0.036).

**Conclusion:**

Older patients with higher creatinine levels, higher Pro/Cr ratios, and lower glomerular filtration rates at disease onset may require more aggressive treatment. Combination therapy with mycophenolate mofetil and corticosteroids yields better outcomes, leading to increased remission rates. These findings provide valuable insights for managing FSGS patients.

Focal segmental glomerulosclerosis (FSGS) is a histological pattern of injury observed on light microscopy (LM) rather than a standalone disease. It is characterized by consolidating a segment of the glomerular capillary tuft connected to Bowman's capsule and accumulating an extracellular matrix involving a subset of glomeruli in the late stages (1, 2).

FSGS is recognized as one of the primary glomerular causes of end-stage kidney disease (ESKD) in most parts of the world. Recent studies have shown that FSGS has become the second most prevalent primary glomerular disorder. Data from various countries consistently reveal FSGS as the most commonly detected glomerulopathy through kidney biopsy (1-6). This increasing trend in FSGS frequency may be attributed to aging and obesity (7). 

While extensive documentation exists regarding the histologic features and clinical characteristics of FSGS patients over the past eight decades, recent focus has shifted towards understanding the disorder's incidence and prevalence across different populations. Earlier studies attempting to elucidate the epidemiological trend of FSGS have demonstrated an increased prevalence in recent years. For instance, FSGS was found in 2.5-4% of native renal biopsies previously, to 12.2-18.7% recently, making FSGS the most common diagnosis based on native kidney biopsies (8). 

Similarly, a recent study in Nepal reported primary FSGS in 10.3% of patients undergoing renal biopsy for glomerular diseases (9). In the United States, FSGS was observed in up to 35% of adults undergoing biopsy to evaluate idiopathic nephrotic syndrome (10).

Identifying epidemiological features, risk factors, and prognostic markers for diseases is crucial for improving management strategies. Primary glomerular diseases have become the predominant biopsy-proven kidney disease, with FSGS being one of the most common glomerular disorders and exhibiting a rising prevalence trend (11, 12). Previous reports have shown that separate administration of mycophenolate mofetil and prednisolone reduces relapse and induces remission (9). Conversely, another study found no correlation between the use of an angiotensin-converting enzyme (ACE) inhibitor or an angiotensin receptor blocker (ARB) and patient outcomes (6). Understanding tailored treatment approaches based on patient characteristics and emphasizing the potential benefits of combined therapy is crucial for achieving remission in FSGS patients.

To our knowledge, no similar study has been conducted in our region. Therefore, the present study aimed to provide an epidemiological report on primary FSGS patients in Shiraz, Iran. The study seeks to understand the demographic characteristics, risk factors, and prognostic indicators of FSGS based on the treatment regimens employed.

## Methods

**Study Population: **This retrospective cohort study enrolled 53 patients with biopsy-confirmed FSGS monitored for five years. The patients were referred to clinics affiliated with Shiraz University of Medical Sciences. The study design involved comparing the medical records of individuals who shared numerous similarities but differed in specific characteristics (e.g., type of treatment, gender, or age) to evaluate outcomes such as remission or relapse. Outpatient visits were scheduled every two months for follow-up assessments.

**Inclusion Criteria: **The inclusion criteria included patients who underwent renal biopsy, were 18 years or older, exhibited proteinuria exceeding 2g/day or >500mg/day with elevated serum creatinine (sCr) levels or hematuria, and were diagnosed with primary FSGS. The study did not exclude cases of obesity, as it could not assess whether obesity was a definitive factor contributing to the disease diagnosis.

**Exclusion Criteria- **The exclusion criteria involved cases with incomplete or insufficient tissue samples obtained from the biopsy, rendering them inadequate for analysis.

**Ethics Approval: **The study received approval from the Ethics Committee of Shiraz University of Medical Sciences, ensuring compliance with ethical guidelines (Ethic No: IR.sums.REC. 1394. S475**)**

**Data Collection: **The study's starting point was the time of the kidney biopsy confirming the disease, and the baseline measurements were taken at the initiation of the study, coinciding with the biopsy. A data-gathering sheet was prepared for each patient, comprising three parts. The first part encompassed comprehensive patient history, including age, gender, diagnosis date, biopsy causes, initial clinical presentation, symptoms, risk factors for secondary FSGS (such as heroin abuse, HIV positivity, family history), and medications.

The second part consisted of laboratory test results during the biopsy and five years later. These tests included white blood cell count, hemoglobin, platelet count, cholesterol level, ANA, anti dsDNA, C3, C4, serum creatinine (sCr), 24-hour urine protein, and creatinine clearance.

The third part focused on histopathological findings, such as tubular atrophy, interstitial fibrosis, mesangial proliferation, FSGS classifications (NOS, Tip, Hilar, Cellular, and Collapsing), as well as disease behavior and patients' prognosis (complete remission, partial remission, activity, relapse, and chronic kidney disease). Most of the specimens were evaluated by the same pathologist.

The required data were collected from patient files at Namazi Hospital, affiliated with Shiraz University of Medical Sciences. For missing data, efforts were made to contact the patients and schedule appointments to gather the necessary information. A trained nurse conducted these appointments and filled in the missing variables. To minimize recall bias, when making appointments to collect missing data, the emphasis was on obtaining demographic information while relying on documented evidence for treatment and medication details. Missing data were handled by omitting variables with significant missing values (e.g., some lab or pathological data) or excluding individuals with incomplete data. There were 62 patients initially, but nine cases had to be omitted due to incomplete data. 

**Definitions and formula: **The creatinine (Cr) clearance (ml/min) was calculated using the following formula: Cr clearance = urine Cr concentration (mg/dL) × 24-hour urine volume (dL) / plasma Cr (mg/dL) × 1440 minutes. The chronic kidney disease (CKD) epidemiology collaboration equation (CKD-EPI) and modification of diet in renal disease study (MDRD) were utilized to calculate the glomerular filtration rate (GFR) (13-14). Samples with more than seven glomeruli in optical microscopy were considered sufficient for reporting (15). Light sheet and fluorescence microscopes were employed for all participants, while transmission electron microscopy was used for some cases. FSGS subtypes were determined based on the Columbia classification, which divided FSGS into five categories: collapsing variant, glomerular tip lesion, cellular, perihilar, and not otherwise specified (NOS). Remission evaluation occurred after 12 to 16 weeks of treatment. Clinically active proteinuria was defined as proteinuria ≥500 mg/day, while complete remission was considered proteinuria ≤300 mg/day after treatment. Partial remission was characterized by a reduction in proteinuria to 300-3500 mg/day and more than a 50% decline from the baseline after treatment. Relapse of FSGS was determined by proteinuria exceeding 3500 mg/day in individuals who previously achieved complete remission or a more than 50% increase in proteinuria in patients who had previously achieved partial remission (16-17). High serum cholesterol level was defined as equal to or greater than 240 mg/dL. Anemia was defined as hemoglobin below 13 g/dL in men and below 12 g/dL in women, while thrombocytopenia was described as a platelet count below 150,000/mm (3).

Treatment for patients with proteinuria < 3g/day commenced with Angiotensin-Converting Enzyme Inhibitors. In cases where proteinuria was >3g/day, Glucocorticoids were added and gradually tapered off after three months until discontinued. If proteinuria increased during or after Glucocorticoid treatment, or if side effects of Glucocorticoids occurred, the first-line treatment option was Cyclosporine or Tacrolimus. For cases where GFR was less than 30 cc/min, Mycophenolate mofetil (MMF) was prescribed (18).

**Ethical considerations: **The study was conducted in accordance with the principles outlined in the Helsinki Declaration of 1975, as revised in 1983. The Ethics Committee of Shiraz University of Medical Sciences approved the research protocol (ethical code: IR.sums.REC. 1394. S475).

**Statistical analysis: **Statistical analysis was performed using Statistical Package for Social Sciences Version 18 (SPSS Inc, Chicago, IL, USA). Continuous variables were presented as mean ± standard deviation (SD), while quantitative or categorical data were presented as frequency and percentage. Changes in laboratory tests over the five years of follow-up were evaluated using paired t-tests and Wilcoxon tests. Furthermore, independent t-tests (Mann-Whitney as a nonparametric test), ANOVA (Kruskal-Wallis H as a nonparametric test), and chi-square tests were employed to assess the relationship between laboratory tests, medications, and prognosis. A significance level of p < 0.05 was considered statistically significant.

## Results

Fifty-three (23 men (43.4%)) patients were included in the study, and their 5-year follow-up data were collected. The mean age of patients at the onset of the disease was 41.0±13.3 years.

Table 1 presents the signs and symptoms observed in these patients. Peripheral edema was the most common manifestation, observed in 30 (56.6%) patients, followed by hypertension in 11 (20.8%) patients. Nephrotic syndrome was the most frequent presentation leading to biopsy, observed in 43 (81.1%) patients. Among the study participants, three (5.7%) patients had a positive family history of renal disease among their first-degree relatives. Histopathological examination of biopsies revealed that tubular atrophy was observed in 28.3% (15 cases), while interstitial fibrosis was present in 24.5% (13 cases). The mean percentage of tubular atrophy and interstitial fibrosis was 13.3±6.9% and 13.8±6.2%, respectively. The most common types of FSGS were "not otherwise specified" (NOS) and tip variant, observed in 7 cases (13.2%) and 4 cases (7.5%) of the biopsies, respectively.

Among the patients, 15 (28.3%) achieved complete remission, while 14 (26.4%) experienced partial remission. Five (9.4%) patients had a relapse of nephrotic syndrome. Chronic kidney disease developed in three (5.7%) patients, and one (1.9%) patient died due to cardiovascular events at the age of 70 years.

Table 2 presents the results of various laboratory tests. Anemia was the most common abnormality detected among FSGS patients, observed in 10 (18.9%) patients, followed by hypercholesterolemia in 7 (13.2%) patients.

**Table 1:** Baseline characteristics of our studied patients

**Characteristics, N=53**		**Mean±SD/Number (%)**
**Age**, Year		41.0±13.3
**Gender**, Men, n (%)		23(43.4)
**Symptoms**	Peripheral edema Hypertension Macroscopic hematuria Others	30(56.6) 11(20.8) 7(13.2) 5(9.4)
**Causes of biopsy**	Nephrotic syndrome Nephritic syndrome Post-transplant	43(81.1) 3(5.7) 3(5.7)
**Positive family history**		3(5.7)
**Histopathology Findings**	Tubular atrophy Interstitial fibrosis Mesangial proliferation	15 (28.3) 13(24.5) 10(18.9)
**Disease behavior**	Complete remission Partial remission Activity Relapse Chronic kidney disease Dead Patients with some data missing	15(28.3) 14(26.4) 9(17.0) 5(9.4) 3(5.7) 1(1.9) 6(11.3)

**Table 2:** The baseline of laboratory tests among our studied group

**Laboratory tests**	**Mean±SD / Number (%)**
**WBC**, per mm3 High low	9329.6±5024.9 3(5.7) 4(7.4)
**Hb, **gr/dl Anemia	13.6±2.2 10(18.9)
**PLT**, per mm3 Thrombocytopenia	239000±81004.6 3(5.7)
**Cholesterol, **mg/dl High	234.9±67.1 7(13.2)
**Positive ANA**	1(1.9)
**Positive Anti dsDNA**	5(9.4)
**Low C3**	0(0.0)
**Low C4**	0(0.0)

SD: Standard deviation, WBC: White blood cells, Hb: Hemoglobin, PLT:  platelet, ANA: Antinuclear. Antibody, DNA: Deoxyribonucleic acid, C3: Complement component 3, C4: Complement component 4.

Table 3 displays the results of kidney function and 24-hour urine tests at the time of biopsy and five years later. The only significant change observed during the study was in the 24-hour urine protein levels, which decreased from 3673.5±8652.6 at the biopsy to 2155.6±2160.6 five years later (P=0.045).

**Factors influencing disease behavior:** sCr, Pro/Cr ratio, MDRD GFR, and CKD-EPI GFR were found to be associated with disease behavior. Patients who achieved remission had lower baseline sCr and Pro/Cr ratios. In contrast, those with active disease had higher levels of baseline sCr (Mann-Whitney, P=0.040) and elevated Pro/Cr ratios (Mann-Whitney, P=0.050). Additionally, patients in remission had higher MDRD GFR and CKD-EPI GFR values at disease onset (Mann-Whitney, P=0.014 and P=0.012, respectively). Age at disease onset was significantly related to disease behavior, with older patients experiencing more disease activity, while remission was more common in younger individuals (Independent t-test, P=0.012). However, no significant association was found between gender and disease prognosis (Chi-square test, P=0.699).

The relationship between the medical regimen and prognosis is presented in table 4. The most frequently prescribed medication was an angiotensin-converting enzyme (ACE) inhibitor or an angiotensin receptor blocker (ARB), which was given to 81.5% of FSGS patients (44 cases). 

Corticosteroids were the second most common treatment, administered to 59.3% of patients (32 cases). The combination of corticosteroids and mycophenolate mofetil (used in 17 patients, 31.5%) was the only treatment regimen that showed a potential association with remission. Corticosteroids and mycophenolate mofetil were prescribed more frequently in patients who achieved remission compared to those who experienced relapse (independent t-test, P=0.036), while neither of these drugs showed a significant relationship with relapse (Independent t-test or Mann-Whitney test, p>0.05). 

**Table 3:** Changes in renal function analysis during the five years

**Laboratory tests**, Mean±SD	**At biopsy**	**Five years later**	**P-value**
**Creatinine**	1.5±1.0	1.3±0.9	0.454
**24hr urine protein**	3673.5±1652.6	2155.6±2160.6	**0.045**
**Protein /** **Creatinine**	4.1±3.7	2.1±2.9	0.396
**MDRD GFR**	71.1±38.7	72.4±24.9	0.399
**CKD epi-GFR**	70.8±34.3	77.5±28.4	0.575
**Creatinine clearance**	100.4±76.5	96.5±40.9	0.262

SD: Standard deviation, GFR: Glomerular filtration rate,‬ MDRD: Modification of Diet in Renal Disease, CKD epi: Chronic kidney disease epidemiology collaboration.

**Table 4:** Drug regimen and its relationship with disease behavior

**Drugs**	**Total** N=53	**Relapse,** n=5		**Remission, **n=29	
		**n (%)**	**P-value **(relaps&not)	**n (%)**	**P-value** (remission&not)
**ACE/ARB**	44(81.5)	4(9.0)	1.000	25(56.8)	1.000
**Corticosteroid**	32(59.3)	2(6.2)	0.625	18(56.3)	1.000
**Mycophenolate mofetil**	28(51.9)	1(3.6)	0.133	18(64.3)	0.249
**Corticosteroid+ Mycophenolate mofetil**	17(31.5)	0(0.0)	0.128	12(70.6)	**0.036**
**Cyclophosphamide**	3(5.6)	0(0.0)	1.000	1(33.3)	1.000
**Calcinurine inhibitor**	15(28.3)	1(6.7)	0.626	8(53.3)	0.423

N: Number, ACE/ARB: Angiotensin converting enzyme inhibitors/angiotensin receptor blockers.

## Discussion

Membranous glomerulonephritis (MGN) and FSGS have emerged as the most prevalent causes of idiopathic glomerular disease (1, 11, 17, 19-20). The primary objective of this study was to examine the demographic and clinical characteristics of patients with FSGS. Additionally, we aimed to evaluate the prognosis of these patients and identify the factors associated with the prognosis.

Our findings indicate that patients who were older at the initiation of treatment displayed a higher susceptibility to disease activity. Moreover, He HG et al.’s study reported that individuals aged 60 and above exhibited a significant risk factor for the progression of FSGS (21). It provides complementary evidence that age significantly influences disease activity and progression among glomerular diseases, specifically FSGS. Older patients may necessitate more vigilant monitoring and tailored interventions to manage disease progression effectively.

In contrast to a previous study that examined the influence of gender on glomerular diseases and reported better outcomes in women, our study yielded different results. We observed no significant differences in the prognosis of men and women with glomerular diseases (22). It is essential to acknowledge that disparities in study design, sample size, patient population, and other variables can contribute to divergent findings between studies. Given these discrepancies, additional research is needed to reconcile these conflicting results and establish a more comprehensive understanding of the impact of gender on the prognosis of glomerular diseases.

Our study observed a direct relationship between baseline sCr levels and the Pro/Cr ratio with disease activity in glomerular diseases. Conversely, the estimated GFR using the MDRD and epidemiologic GFR estimation methods demonstrated an inverse correlation with disease activity. These findings are consistent with previous studies (6, 17, 23-24). Higher sCr levels and lower GFR indicate greater renal inflammation and structural damage in glomerular diseases. Increased inflammation can contribute to disease activity, while heightened scarring and fibrosis impair kidney function, resulting in a worse prognosis. A study by Korbet et al. suggested that a serum creatinine level above 1.3 mg/dl is a predictive value for developing end-stage renal disease (ESRD) (17). The association between higher sCr and lower GFR at initiating FSGS reflects significant kidney dysfunction, advanced disease severity, renal inflammation, scarring, and reduced renal reserve. These factors collectively contribute to increased disease activity and indicate a poorer prognosis for individuals with FSGS. Most previous studies have reported that the histopathological type of FSGS has predictive value. However, in our study, the results of the histopathological type of our patients were not available, preventing us from evaluating this factor. Although we did not find a correlation between disease behavior and interstitial fibrosis or tubular atrophy, some previous studies have suggested that less severe tubulointerstitial injury is associated with a higher likelihood of remission (6, 17). Furthermore, these studies have indicated that more than 20% of interstitial fibrosis predicts end-stage renal disease (ESRD) (17).

The confounding factor in our study, which did not demonstrate a correlation between tubular atrophy and interstitial fibrosis with prognosis, is the lack of information regarding the histopathological type of FSGS. It is well known that FSGS can have different histopathological subtypes, which can have varying prognostic implications. The absence of histopathological type information in our study prevents us from accounting for the potential influence of different subtypes on the relationship between tubular atrophy, interstitial fibrosis, and disease prognosis. This missing information may confound the results and limit our ability to draw definitive conclusions regarding the correlation between tubular atrophy, interstitial fibrosis, and disease behavior. Prednisolone is commonly recommended as the primary choice for immunosuppressive treatment, either as a standalone medication or in combination with other immunosuppressants (17). Previous studies have shown that separate administration of mycophenolate mofetil and prednisolone can reduce the relapse rate and induce remission, respectively (9, 25-27). The combination of corticosteroids and mycophenolate mofetil has demonstrated a higher remission rate among various drug regimens prescribed to our patients. Importantly, achieving remission, irrespective of histopathologic types or other factors, indicates a favorable outcome (17, 25-29). Consequently, the concurrent use of corticosteroids and mycophenolate mofetil has yielded positive outcomes in our study, aligning with findings from another study by Thomas et al. (6). Thomas et al. (6) found no correlation between the use of angiotensin-converting enzyme (ACE) inhibitors or angiotensin receptor blockers (ARBs) and patient outcomes in our study or the study. However, these medications have been suggested as preventive measures for proteinuria, indirectly slowing down the progression of renal impairment (29-30). Korbet et al. have also recommended ACE inhibitors and ARBs as conservative and initial therapies for FSGS (17). Consequently, they were the most commonly prescribed drugs among our patients.

This study provides insights into the demographic characteristics, risk factors, and prognostic indicators of FSGS in Shiraz, Iran. Our findings highlight the effectiveness of combined therapy involving steroids and mycophenolate mofetil in inducing remission among our patients. However, further research is necessary to investigate the risk and prognostic factors associated with FSGS and determine the optimal treatment approach for these patients.The limitations of this study stem from its epidemiological report and retrospective design, as well as the absence of a registry system specifically addressing this issue in our center. Additionally, the study was conducted at a single center with a small sample size, which may limit the generalizability of the findings. We recommend conducting further studies on this subject with a multi-center approach and larger sample sizes to overcome these limitations. It is essential to acknowledge that various confounding factors, including concomitant diseases, may have influenced the treatment response of our patients. Furthermore, the lack of genetic workup prevented the confirmation of familial cases, potentially affecting the response to treatment and achievement of remission.
